# Stability and cross-lagged relations among callous-unemotional traits, moral identity, moral emotion attribution and externalizing behavior problems in adolescents

**DOI:** 10.1007/s12144-023-04755-2

**Published:** 2023-05-19

**Authors:** Neele Bäker

**Affiliations:** School of Educational and Social Sciences, Department of Special Needs Education and Rehabilitation, Carl von Ossietzky University of Oldenburg, Ammerländer Heerstraße 114, 26129 Oldenburg, Germany

**Keywords:** CU-traits, Moral identity, Moral emotion attribution and externalizing behavior problems, Adolescence, Longitudinal design

## Abstract

This study investigates the associations of adolescents callous-unemotional traits with moral constructs and the interplay of various outcomes. The present study builds on the lack of research and focuses on the longitudinal relationships between CU-traits, moral identity, moral emotion attribution and externalizing behavior problems in adolescence. The included variables were collected at test time points T1 and T2. To determine the predictive, and stability links among the variables, a cross-lagged model in SPSS AMOS 26 was conducted. Time stability path estimates were moderate to highly stable over time for all included variables. Significant cross lagged paths of moral identity_T1_ on moral emotion attribution_T2_, CU-traits_T1_ on moral identity_T2_, externalizing behavior problems_T1_ on moral emotion attributions_T2_ and externalizing behavior problems_T1_ on CU-traits_T2_, could be found.

## Introduction

Callous-unemotional traits are a well-established empirical predictor of aggressive and delinquent behavior. Yet little is known about how the relationship between CU-Traits and moral development affects externalizing behavior problems. Callous-unemotional (CU) refers to a constellation of personality traits leading to more persistent and aggressive types of antisocial behavior in adolescents (Frick et al., 2003). Currently it is not clear whether any, and if they are, which facets of the moral decision-making process are influenced by CU-traits or vice versa. There are several moral constructs that can be noted in this context, such as moral identity or moral emotions. Moral identity is understood as part of the self-concept that leads to moral behavior (Aquino & Reed, 2002). Stets and Carter (2011) exposed that discrepancies between individual’s moral identity and behavior are associated with negative self-evaluative emotions. In the current study it was investigated whether the interplay between risk factors like CU-traits and protective factors like moral identity and moral emotion attributions can determine the occurrence and severity for externalizing behavior and whether these constructs show stability in development during adolescence.

## Theoretical background

CU-traits in childhood and adolescence are named as precursors of psychopathy in adulthood developing a concept of psychopathy in childhood and adolescence (Essau et al., 2006; Koglin & Petermann, 2012). Affective-social deficits such as low empathy, lack of remorse for wrongdoing, emotional expression, and concern about one’s own performance or the feelings of others were denoted (Frick et al., 2003; Koglin & Petermann, 2012). CU-traits can disrupt the development of conscience and increase aggression and strong antisocial behavior (Frick et al., 2014). CU-traits are thought to be high in 2–7% of children and adolescents without conduct disorder (Kahn et al., 2012). 10–32% of children and adolescents show high levels of CU-traits when conduct disorder is observed (Kahn et al., 2012). From a psychological perspective, CU-traits stability, and their influence on development in childhood and adolescence is of particular importance. Up to now, research has not been able to clarify from which age on the concept of CU-traits can be assessed and used meaningfully (Koglin & Petermann, 2012). Frick et al. (2014) addressed the question of CU-traits stability in a review and found stability differences across reporters, with a rather substantial level of stability across childhood and adolescence. Furthermore, they highlight the importance of considering the level of stability in CU-traits from childhood or adolescence to adulthood compared to the stability of other psychopathological constructs.

Moral identity is defined by how important moral qualities are to the self-concept (Hardy & Carlo, 2011). Research indicates that high centrality of moral characteristics for defining one’s self is positively associated with prosocial behavior and negatively associated with aggressive and antisocial behavior (Hardy et al., 2015; Hertz & Krettenauer, 2016). A strong moral identity motivates people to act in accordance with their moral values (Oser, 2013). Moral identity manifests in middle childhood (Kingsford et al., 2021; Krettenauer et al., 2013). The compliance to moral norms becomes more internally motivated in this age range (Krettenauer & Hertz, 2015). According to Matsuba et al. (2014), there are many different factors that contribute to the creation of a moral identity, for example, personality traits.

Furthermore, Oser (2013) describes moral emotions attributions as a part of the moral motivation for moral action. For example, moral emotions play a major role in the moral development process determining how children and adolescents evaluate moral conflicts (Tangney et al., 2007). A common use of anticipated moral emotions are emotion attributions in hypothetical moral rule-breaking or hypothetical moral dilemmas (Lagattuta, 2005). Based on this, either a positive (happy victimizer response pattern) or negative emotion attributions pattern (unhappy victimizer response pattern) develop after an action (Lagattuta, 2005). Moral emotion attributions were consistently associated with more prosocial and less antisocial behavior serving as an indicator for behavior (Malti & Krettenauer, 2013). However, feeling ‘negative’ emotions after wrongdoing is not generally expected. Specifically, aggressive adolescents are less likely to feel ‘negative’ emotions after rule transfers and more likely to express positive emotions than ‘non-aggressive’ adolescents (Arsenio et al., 2004; Malti et al., 2009). It is reasonable to assume that a lack of emotions, due to personality traits, expresses a lack of prosocial behavior.

All three constructs, CU-traits, moral identity, and moral emotion attributions, are related to pro- and antisocial behavior (Frick & Viding, 2009; Hardy et al., 2015; Hertz & Krettenauer, 2016; Malti & Krettenauer, 2013). Based on the theoretical derivation, it can be concluded that a lack of emotions, due to personality traits, expresses a lack of prosocial behavior and encourages an increase of externalizing behavior problems.

## Current study

The present study examines longitudinal relationships between CU-traits, moral identity, moral emotion attribution and externalizing behavior problems in adolescence. In particular, there is a lack of studies that examine older children, especially adolescents and combine moral constructs such as moral identity and moral emotion attributions with callous-unemotional traits and externalizing behavior problems and control adolescents age and gender. While several associations have been studied e.g. in relation to CU-traits and behavioral constructs (Frick & Viding, 2009; Goagoses & Schipper, 2021; Kochanska et al., 2013; Koglin & Petermann, 2012), moral constructs, especially moral identity and moral emotion attribution, have received less attention in this context. Because high CU-traits affect only a small proportion of children and adolescents (Kahn et al., 2012), analyses were included that examined all scales for high and low CU-traits. Although from a developmental point of view, it can be assumed that CU-traits show an influence on behavior (Frick & Viding, 2009; Kochanska et al., 2013; Koglin & Petermann, 2012) studies suggest that it can also be assumed that reciprocal effects can be found with behavior disorders (e.g. Brown et al., 2017). Considering the presented studies, the following conclusions can be drawn: Empirical correlations between the constructs could be raised. The current state of studies is very poor in all areas. The studies on moral identity and the behavior of adolescents are mainly based on the prosocial or moral behavior of adolescents; externalizing behavior problems are neglected in current research. CU-traits have been increasingly analyzed in the context of moral disengagement, but not in the context of moral identity. Recent studies show that high moral identity in adolescents is associated with lower levels of CU-traits (Schipper & Koglin, 2021a). This direction of correlations is consistent with the assumption that CU-traits are characterized by e.g. callous attitudes towards other persons, which manifests itself in a lack of empathy, guilt, and remorse for wrongdoing (Essau et al., 2006). These traits are not consistent with a high moral identity, characterized by individuals rating traits such as “It is important to me to be a compassionate person” as high (Aquino & Reed, 2002; Koglin, 2017) so that a negative relationship is assumed. Furthermore, it was shown that a high moral identity is negatively related to positive emotions after rule breaking (Schipper & Koglin, 2021b). For the present study, positive emotion attributions were queried following a moral transgression. From theory, it can be inferred that individuals feel bad after moral transgressions. Therefore, it is hypothesized that moral identity is negatively related to moral emotion attributions. In addition, negative associations between high moral identity with behavior problems can be inferred from recent studies (Hardy et al., 2015; Hertz & Krettenauer, 2016). Accordingly, a high moral identity would not be consistent with harmful “immoral” actions, so a negative link between moral identity and externalizing behaviors is assumed.

Longitudinal cross-lagged panel path analyzes were conducted to test the (a) stability in the variables over time, (b) correlations between variables within each time, and.

(c) the cross-lagged relations between variables over time. The central research question was: Which longitudinal cross lagged and stability paths are shown between the constructs of personality, morality and behavior? The aims of the current study are (a) to examine the longitudinal stability in CU-traits, moral identity, moral emotion attribution and externalizing behavior problems and (b) to test the longitudinal relationships between these constructs, providing additional evidence for possible longitudinal predictors of externalizing behavior problems. It was expected that all time stability paths have medium stability, further it was hypothesized that:

- Moral identity is negatively correlated with CU-traits, moral emotion attributions (after rule breaking) and externalizing behavior problems.
- CU-traits show a longitudinally influence on moral identity and moral emotion attribution later times.
- Moral identity will be longitudinally associated with moral emotion attributions later times.
- CU-traits, moral identity and moral emotion attributions have a significant longitudinally influence on externalizing behavior problems later times.
- There are reciprocal associations between CU-traits and externalizing behavior problems in adolescence.

Adolescence is the developmental stage that precedes entry into adulthood. Adolescence represents a phase of life from late childhood to adulthood. The ages used to define this phase vary. However, the start of puberty and the beginning of adulthood are often used to delineate this phase (Silbereisen & Hasselhorn, 2008). In terms of developmental theory, adolescence is associated with developmental tasks, such as identity development and tasks in the area of social relationships (Silbereisen & Hasselhorn, 2008). The developmental stage is characterized by rapid biological, psychological, and relational changes that directly affect the development of personality traits. Adolescence therefore represents a critical period for personality development (Slobodskaya & Kornienko, 2021). By focusing on adolescence, the study was drawn from a longitudinal (two time points), based study. Furthermore, the study refers to adolescence, as in this age range, the sense of moral identity and related stability in moral action tendencies are established (Keller, 2004). An important research gap that still exists is the lack of analysis of the stability of moral constructs like moral identity and moral emotion attributions as well as the associations with CU-traits and how these constructs lead to behavior problems. A novelty is that the data relevant to this study are available for each variable at two test time points. Due to the limited number of studies the study is of an exploratory nature.

## Methods

### Participants and procedure

The data stems from a lager project led by the author analyzing social-emotional and moral development of adolescents. Only instruments and data relevant to the current research questions are reported. 17 schools have agreed to participate in the study. A total of *N* = 750 adolescents participated in the first wave. *N* = 632 adolescents participated in the second survey wave. Of these, *n* = 130 adolescents could be linked to the data from T1. Accordingly, longitudinal data from 130 students are available for this study. Due to data privacy policies the names of the schools could not be recorded therefore students that did not state their full name could not be linked. Additionally, participation for schools and students was voluntary therefore some schools and students decided not to participate in the second wave. A power of 0.95 was achieved with a post hoc power analysis with G*Power and detected an effect of *f*^*2*^ = .05 with α = 0.05 (Faul et al., 2007, 2009) the sample was sufficient for the present analyses. Table 1 shows the descriptive statistics for the study participants. The sample included 65 females and 65 males, with ages ranging from 10 to 17 (T1) and 11 to 18 (T2) (*M*_T1_ = 13.88, *M*_T2_ = 14.82; *SD*_T1_ = 1.26, *SD*_T2_ = 1.29). The Gender distribution of the sample is homogeneous. The adolescents attend various types of secondary schools, including those that are more academically focused (Gymnasium; 24.6%) and those that are practically focused (Oberschule; 75.4%). The majority were born in Germany (86.2%) and reported that German was their mother tongue (74.6%). Through the teachers, it was ensured that the participating students had sufficient knowledge of German to take part in the study. Students who do not have sufficient knowledge of German were excluded from the study.

**Table 1:** Descriptive Statistics for age, school type and socio-economic status

	Girls	Boys
Age Group		
< = 14	48	47
> 14	17	18
School Type		
Academically Focused	17	17
Practically Focused	48	48
SES		
Born in Germany	56	56
Mother Tongue (German/Other)	44/21	53/12

Median split for Age T1 (14 years); SES = socio-economic status

The study has received a positive vote from the Commission for Research Assessment and Ethics and the authors declare no conflicts of interest. In addition, the study was reviewed by the Data Protection Authority of the University where this study was conducted, and the approval of the responsible State Education Authority were obtained. At first, schools were recruited. In case of interest to participate organizational procedures were carried out, such as sending out the general participant information and obtaining the consent forms for the implementation of data collection by the school principals.

The data was collected at two measurement times. The first test time was in spring 2020 (T1). The second measurement time was in spring 2021 (T2). Due to the COVID-19 pandemic containment measures, the survey of students was organized as an online survey with *LimeSurvey*. The selection of schools was made randomly. If school administrators agreed to participate in the study, the information was shared with teachers and subsequently with students and their caregivers. Schools were encouraged to provide the information to students and caregivers either via email or the school’s internal server. All relevant information for the participants, as well as the consent forms, were included in the links for the study and were provided or requested prior to the start of the survey. Only after confirmation of consent the questionnaire was made accessible. Since participation was voluntary and required the informed consent of both, the caregivers and the children, it was not possible to ensure that entire classes could be surveyed. If not all consents were obtained, the students were excluded from the study. The students did not receive any compensation.

### Instruments

#### Moral identity

Koglin and Daseking (2017) developed 18 items to capture moral identity, through which, the participants were asked to assess the subjective importance of 18 human characteristics. Based on The Self-Importance of Moral Identity (Aquino & Reed, 2002), nine items measured moral identity (for example “compassionate”, “trustful”). Based on the adapted version of the Good Self-Assessment (Barriga et al., 2001), the nine moral characteristics were supplemented by nine “non-moral” characteristics as distractors (for example “popular”, “ambitious”). Participants responded the subjective importance of these characteristics on a scale ranging from (1) not important to me to (4) very important to me (*α*_T1_ = .78, *α*_T2_ = .71; 9 items). The moral identity scale was formed by summing the 9 moral identity items. Higher scores indicating higher moral identity levels. The structure of moral identity was tested using exploratory factor analysis. Both Bartlett’s test (Chi-Square(36) = 272.69, p < .001) and the Kaiser-Meyer-Olkin Measure of Sampling Adequacy (KMO = .770) indicate that the variables are suitable for factor analysis. Thus, a principal component analysis was conducted. It is found that all items load between .34 and .78 onto the factor.

#### Moral emotion attribution

The emotion attribution comprised 12 short stories based on Weller and Lagattuta’s moral dilemmas (2012, 2014), showing moral conflict situations, in which the protagonist is pushed in two different directions by incompatible moral reasons (Christensen & Gomila, 2012). Transgressors and victims were matched to the participant’s age and gender. Anticipated emotions were assessed using a measure consisting of hypothetical moral dilemmas. The adolescents stated how good or bad the protagonist felt when not helping the other person. For example (girls’ version), ‘Lisa decides to push/hit Jennifer. How good or bad do you think Lisa feels about it?’ Answers were given on a scale ranging from (1) very good to (5) very bad (*α*_T1_ = .82, *α*_T2_ = .81; 12 items; (Koglin & Daseking, 2017; Schipper & Koglin, 2021b). Mean and sum scores for the overall measure were calculated, higher scores indicating feeling good by not helping.

The *Inventory of Callous-Unemotional Traits* (Essau et al., 2006; Kimonis et al., 2008) was used to assess callous-unemotional traits. Adolescents respond on a scale ranging from (1) not at all true to (4) definitely true. The questionnaire includes three subscale (1) *callousness* (9 items, e.g., ‘I do not feel remorseful when I do something wrong’), (2) *unemotional* (5 items, e.g., ‘I do not show my emotions to others’), and (3) *uncaring* (8 items, e.g., ‘I try not to hurt others’ feelings’ – reverse coded) (*α*_T1_ = .81, *α*_T2_ = .78; 24 items). Mean and sum scores for the overall CU measure were calculated, higher scores indicating higher CU-traits levels.

#### Externalizing behavior problems

To assess externalizing behavior problems, adolescents completed the *Youth Self-Report* checklist (Döpfner et al., 2014; Child Behavior Checklist, Achenbach, 1991). On a scale ranging from (0) not true to (2) very or often true, adolescents stated how often they displayed certain behaviors as ‘I break rules at home, at school, or anywhere else’ in the past six months (*α*_T1_ = .88, *α*_T2_ = .88; 32 items). For the current study, the subscale rule- breaking (e.g., lying, stealing) and aggressive behaviour (e.g., property damage, fighting) were summed up. Higher scores indicating higher externalizing behavior problems.

### Data analytic procedures

SPSS Statistics 27 and SPSS AMOS 26 were used to analyze the collected data. The included variables were collected at test time points T1 and T2. Calculation of descriptive analysis, Cronbach’s Alpha, correlation analysis, and ANOVA with repeated measurement for differences in T1 and T2 and interaction for gender and age were conducted in SPSS Statistics version 27. To control the variables for the expression of the CU-traits a MANOVA (SPSS Statistics 27) for high and low CU-Traits for all included study variables and time points was used. To determine the predictive, and stability links among the variables, a cross-lagged model in SPSS AMOS 26 was conducted. Because the data were missing completely at random (Little’s MCAR test χ^2^ = 115.58, df = 122, *p* = .647) it was allowed to impute with information maximum likelihood estimator of the regression coefficient in the data analysis. The model fit was evaluated by means of the root means square error of approximation (RMSEA) (Browne & Cudeck, 1993), the comparative fit index (CFI), normed fit index (NFI) and the tucker lewis index (TLI), (Arbuckle, 2012).

## Results

### Preliminary analysis

Descriptive statistics per variable, as well as the correlations between them are provided in Table 2. Results showed significant stability over time. In addition, all variables within each time were positively correlated with each other excluding externalizing behavior problems_T2_ with both moral constructs, moral identity_T1_, moral identity_T2_ and moral emotion attributions_T1_. As expected, moral identity was significantly negative correlated with moral emotion attribution, CU-traits and externalizing behavior problems at both time points. Moreover, CU-traits showed the strongest significant correlations with all included variables. Adolescent gender was only related to moral identity_T1_. Adolescent age indicates correlations with externalizing behavior problems_T1_ and externalizing behavior problems_T2_.

**Table 2:** Descriptive statistics for all study variables and intercorrelations between the study variables

	1.	2.	3.	4.	5.	6.	7.	8.	9.	10.	11.
1. Moral Identity T1	1										
2. Moral Identity T2	.53**	1									
3. Moral Emotion Attribution T1	−.20*	−.25**	1								
4. Moral Emotion Attribution T2	−.44**	−.27**	.52**	1							
5. CU-Traits T1	−.49**	−.51**	.51**	.50**	1						
6. CU-Traits T2	−.27**	−.53**	.40**	.43**	.67**	1					
7. Externalizing Behavior Problems T1	−.19*	−.19*	.27**	.44**	.46**	.44**	1				
8. Externalizing Behavior Problems T2	−.13	−.16	.13	.32**	.19*	.40**	.60**	1			
9. Age T1	−.04	−.06	.00	.11	.04	.03	.26**	.23**	1		
10. Age T2	−.01	.02	.01	.09	.02	.00	.20*	.20*	.95**	1	
11. Gender	−.28**	−.14	−.03	.13	.04	−.01	−.07	−.13	.02	.02	1
* M*	30.43	29.67	26.40	27.56	47.69	49.24	10.59	12.46	13.88	14.82	
* SD*	3.33	3.02	5.75	6.13	7.49	7.16	7.54	7.77	1.26	1.28	
* MD*	30.00	30.00	26.00	28.00	47.00	49.00	10.00	12.00			
* a*	.78	.71	.82	.81	.81	.78	.88	.88			

*p < .05; **p < .01; ***p < .001; *M* = Mean; *SD* = Standard deviation; *MD* = Median

Table 3 presents the ANOVA with repeated measurement for differences in T1 and T2. It can be noted from the table that all constructs changed significantly from T1 to T2.

**Table 3:** ANOVA with repeated measurement for differences in T1 and T2

	T1		T2			Main effect		Interaction with Gender		Interaction with Age	
	*M*	*SD*	*M*	*SD*	*df*	*F*	*η*^*2*^_*p*_	*F*	*η*^*2*^_*p*_	*F*	*η*^*2*^_*p*_
Moral Identity	30.43	3.33	29.67	3.02	1, 126	8.96**	.066	1.58	.012	1.10	.009
Moral Emotion Attribution	26.40	5.75	27.56	6.13	1, 126	6.64**	.050	3.46	.027	1.53	.012
CU-Traits	47.69	7.49	49.24	7.16	1, 126	7.91**	.059	.000	.000	.202	.002
Externalizing Behavior Problems	10.59	7.54	12.46	7.77	1, 126	5.29*	.040	.251	.002	.951	.007

*M* = Mean; *SD* = Standard deviation; *p < .05; **p < .01; ***p < .001; *F* = F-Test, *η*^*2*^_*p*_ = partial eta-square; controlling for gender, median split for Age T1 (14 years)

The results of the MANOVA for high and low CU-traits highlighted significant effects for all included variables and time points (Table 4). All effects show the highest significance level except moral identity at T1 and the distinction for high and low CU traits for T2 as well as for externalizing behavior problems at T2 and the distinction for high and low CU traits at T1. The strongest effects are shown for moral identity, moral emotion attribution and CU-traits whereas the weakest effect is shown for externalizing behavior problems.

**Table 4:** MANOVA for high and low CU-Traits for all included study variables and time points

	T1							T2						
	CU-Traits high		CU-Traits low		Main effect			CU-Traits high		CU-Traits low		Main effect		
	*M*	*SD*	*M*	*SD*	*df*	*F*	*η*^*2*^_*p*_	*M*	*SD*	*M*	*SD*	*df*	*F*	*η*^*2*^_*p*_
Moral Identity T1	28.67	3.43	31.72	2.60	1, 128	32.99***	.205	29.85	3.40	31.13	3.13	1, 128	4.93*	.037
Moral Identity T2	28.33	2.60	30.64	2.94	1, 128	21.76***	.145	28.57	2.88	30.98	2.64	1, 128	24.44***	.160
Moral Emotion Attribution T1	29.45	5.12	24.17	5.16	1, 128	33.54***	.208	28.26	5.06	24.17	5.78	1, 128	18.54***	.127
Moral Emotion Attribution T2	30.33	5.60	25.52	5.71	1, 128	22.89***	.152	30.10	5.26	24.49	5.71	1, 128	33.89***	.209
Externalizing Behavior Problems T1	14.49	8.07	7.74	5.68	1, 128	31.34***	.197	13.97	7.55	6.53	5.21	1, 128	41.03***	.243
Externalizing Behavior Problems T2	14.35	8.14	11.08	7.24	1, 128	5.83*	.044	15.01	8.19	9.41	6.00	1, 128	19.07***	.130

*M* = Mean; *SD* = Standard deviation; *p < .05; **p < .01; ***p < .001; *F* = F-Test, *η*^*2*^_*p*_ = partial eta-square; median split CU-Traits (high and low)

### Stability and cross-lagged relations

**Fig. 1:**
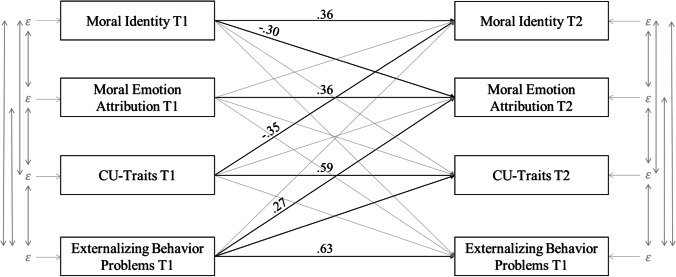
Stability and cross-lagged associations between all included variables. Note: Age and gender were excluded for clarity; Labeled paths are significant at ***p < .001; Fit values (*N* = 130; *χ2/df* = .993, *p* = .470, *CFI* = 1.000, *NFI* = .971, *TLI* = 1.00, *RMSEA* = .000)

To examine the longitudinal associations between CU-traits, moral identity, moral emotion attribution and externalizing behavior problems, stability and cross-lagged analysis were conducted. Results are presented in Fig. 1. The stability cross-lagged panel model is based on all available data from all participants (*N* = 130). Fit statistics of the four full sample cross-lagged models indicated good model fits (*N* = 130; *χ2/df* = .993, *p* = .470, *CFI* = 1.000, *NFI* = .971, *TLI* = 1.00, *RMSEA* = .000). Table 5 shows the standardized path coefficients including time stability paths, cross-lagged paths and covariances for all included variables. Time stability path estimates were moderate to highly stable over time (*ß* = .36; *ß* = .36; *ß* = .59; *ß* = .63, *p* < .001). Significant cross lagged paths of moral identity_T1_ on moral emotion attribution_T2_ (*ß* = −.30, *p* < .001), CU-traits_T1_ on moral identity_T2_ (*ß* = −.35, *p* < .001), externalizing behavior problems_T1_ on moral emotion attributions_T2_ (*ß* = .27, *p* < .001) and externalizing behavior problems_T1_ on CU-traits_T2_ (*ß* = .16, *p* < .05) can be inferred from the table. For each significant control variable, the interaction is examined in the cross-lagged panel analysis (Table 5). Overall, the variance in variables at T2 accounted for by the predictors varied from 36% to 48% in the full sample. Moral identity_T2_ shows an explained variance of 36%, moral emotion attribution_T2_ shows an explained variance of 46%, 48% of the variance is resolved in CU-traits_T2_, and 39% of the variance is resolved in the externalizing behavior problems_T2_.

**Table 5:** Standardized path coefficients

Time stability paths	Estimates
Moral Identity T1 ➔ Moral Identity T2	.36***
Moral Emotion Attribution T1 ➔ Moral Emotion Attribution T2	.36***
CU-Traits T1 ➔ CU-Traits T2	.59***
Externalizing Behavior Problems T1 ➔ Externalizing Behavior Problems T2	.63***
Cross-lagged paths	
Moral Identity T1 ➔ Moral Emotion Attribution T2	−.30***
Moral Identity T1 ➔ CU-Traits T2	.06
Moral Identity T1 ➔ Externalizing Behavior Problems T2	−.08
Moral Emotion Attribution T1 ➔ Moral Identity T2	−.01
Moral Emotion Attribution T1 ➔ CU-Traits T2	.07
Moral Emotion Attribution T1 ➔ Externalizing Behavior Problems T2	.02
CU-Traits T1 ➔ Moral Identity T2	−.35***
CU-Traits T1 ➔ Moral Emotion Attribution T2	.04
CU-Traits T1 ➔ Externalizing Behavior Problems T2	−.16
Externalizing Behavior Problems T1 ➔ Moral Identity T2	.06
Externalizing Behavior Problems T1 ➔ Moral Emotion Attribution T2	.27***
Externalizing Behavior Problems T1 ➔ CU-Traits T2	.16*
Age T1 ➔ Externalizing Behavior Problems T1	.24***
Age T2 ➔ Externalizing Behavior Problems T2	.09
Gender ➔ Moral Identity T1	−.26***
Covariances	
𝜀 Moral Identity T1 ⟷ 𝜀 Moral Emotion Attribution T1	−.22*
𝜀 Moral Identity T1 ⟷ 𝜀 CU-Traits T1	−.50***
𝜀 Moral Identity T1 ⟷ 𝜀 Externalizing Behavior Problems T1	−.22*
𝜀 Moral Emotion Attribution T1 ⟷ 𝜀 CU-Traits T1	.50***
𝜀 Moral Emotion Attribution T1 ⟷ 𝜀 Externalizing Behavior Problems T1	.28**
𝜀 CU-Traits T1 ⟷ 𝜀 Externalizing Behavior Problems T1	.47***
𝜀 Moral Identity T2 ⟷ 𝜀 Moral Emotion Attribution T2	.09
𝜀 Moral Identity T2 ⟷ 𝜀 CU-Traits T2	−.37***
𝜀 Moral Identity T2 ⟷ 𝜀 Externalizing Behavior Problems T2	−.13
𝜀 Moral Emotion Attribution T2 ⟷ 𝜀 CU-Traits T2	.11
𝜀 Moral Emotion Attribution T2 ⟷ 𝜀 Externalizing Behavior Problems T2	.11
𝜀 CU-Traits T2 ⟷ 𝜀 Externalizing Behavior Problems T2	.35***
Age T1 ⟷ Age T2	.95***

*p < .05; **p < .01; ***p < .001; Fit values (*N* = 130; *χ2/df =* .993, *p* = .470, *CFI* = 1.000, *NFI* = .971, *TLI* = 1.00, *RMSEA* = .000)

## Discussion

The purpose of the current study was to examine the stability and cross-lagged associations between CU-traits, moral identity, moral emotion attributions, and externalizing behavior problems among adolescents. Thus, the present study added to the growing body of research on moral development in this age range and extended the knowledge of the stability of CU-traits and moral constructs, as well as externalizing behavioral problems.

In the literature, the relationship between identity and personality constructs has been studied in both theoretical and empirical ways. Overall, research on the stability of CU-traits in antisocial adolescents shows inconsistent results. In this study, time stability path estimates detected moderate to high effects over time for all included variables. Externalizing behavior problems as well as CU-traits show more stability than the moral constructs. The strongest stability is shown by externalizing behavior problems, followed by CU-traits, moral identity and moral emotion attributions. The results depict that all of the present moral constructs continue to change over time and are in line with previous research and theoretical assumptions, describing that identity traits further changing in adolescence (Topolewska-Siedzik & Cieciuch, 2018). In the field of moral research, moral identity is described as a source of moral emotions and actions that evolves over the life course, particularly from childhood to adolescence (Kingsford et al., 2021; Oser, 2013). Moreover, the results of this study can already show moderate stability effects for the moral constructs, this is in line with developmental theoretical approaches that highlight the development of one’s own value system as a task during adolescence. It is assumed that adolescents establish their own moral values during the developmental phase and internalize them as their moral identity (Hardy & Carlo, 2011). In contrast pathological personality traits have been described as stable constructs in recent research (Krischer et al., 2012). Results from studies in childhood also point to these tendencies (e.g., Lynam et al., 2009; Jiang et al., 2022). Although these results were to be expected, the stabilities for CU traits and externalizing behavior problems are alarmingly high. The results extend the current body of research by showing that the constructs remain stable even when controlling for different levels of personality, identity, emotion, and behavior.

It has been suggested that the constructs change during the development of adolescents. From the ANOVA for the constructs at both test time points, it can be seen that the adolescents’ moral identity decreased over time, adolescents felt better about themselves after a rule transition at the second test time point, CU-traits increased, and they showed more externalizing behavior problems. The negative trend in externalizing behavior problems is not consistent with current studies, which describe externalizing behavior problems as declining with increasing age (Krischer et al., 2012) and the general declining trend in Germany (Klipker et al., 2018). This negative development should not only be considered from the aspect of adolescence but should furthermore be viewed critically under the conditions of the Corona Pandemic. A possible explanation for this negative development could be the low personal contact and interactions with peers, emotional distress, and decreases in life satisfaction during the Corona Pandemic (Magson et al., 2021). Even outside of considerations of the pandemic situation, studies show that decreases in life satisfaction (e.g., Zhu & Shek, 2021) are related to increases in behavioral problems.

To overcome the weakness that adolescents are low in their CU-traits, the adolescents were divided into two groups, distinguishing between adolescents with high scores and adolescents with low scores. The results of the MANOVA for high and low CU-traits highlighted significant effects for all included study variables at both time points (Table 4). The development of all included constructs depends on the expression of the CU-traits. The formation of a moral identity is significantly lower with high CU-traits, emotion attributions are significantly higher with high CU-traits (they feel better about immoral acts), and externalizing behavior problems are also significantly higher with high expression of CU-traits. The outcomes pointed out that adolescents with CU-traits showed differences in the expressions of moral identity, moral emotion attributions, and externalizing behavior problems. These results underline that the high expression of personality traits, such as those of the CU-traits, has a significant effect on the development of moral constructs as well as on behavior.

The panel design used in the present study is a rigorous method to establish the causality and reciprocity of the relationships between the present constructs. Significant cross-lagged paths of moral identity_T1_ on moral emotion attribution_T2_, CU-traits_T1_ on moral identity_T2_, externalizing behavior problems_T1_ on moral emotion attributions_T2_ and externalizing behavior problems_T1_ on CU-traits_T2_ could be found.

For the panel design, it was hypothesized that moral identity will be longitudinally associated with moral emotion attributions. A high moral identity shows a significant influence on moral emotions. The higher the level of moral identity, the worse adolescents feel after breaking the rules. This may provide evidence that a person’s internalized norms influence emotions and are thus indicators of moral identity. The results are consistent with the path analysis of Schipper and Koglin (2021b), in which the heuristic model of Oser (2013) was empirically tested and moral identity was named as the central point of moral motivation.

Furthermore, it was hypothesized that CU-traits show a longitudinal influence on moral identity and moral emotion attribution. Moral identity is significantly influenced by CU-traits. The higher the CU- traits the lower the expressions of moral identity. These findings speak to the influence of personality traits on identity and are in line with the findings of Schipper and Koglin (2021a). In order to prevent serious development problems the relevance for preventive interventions is emphasized by Frick and White (2008). These should aim to address the specific characteristics of CU-traits, such as promoting the development of empathy and consideration for others, even before aggression and behavior problems have become severe. The hypothesis that high CU-traits also predict high emotion attributions could not be confirmed.

It was hypothesized that CU-traits, moral identity, and moral emotion attributions have a significant longitudinal influence on externalizing behavior problems later in life. A derivation from theoretical presuppositions led us to assume that the stability of personality traits favors behavioral problems, which in turn favor or reinforce emotions and CU-traits in adolescence. The present results indicated that youth externalizing behavior problems influenced moral emotions as well as CU-traits. Contrary to our expectations, the results of this study did not support the hypothesis that CU-traits, moral identity, and moral emotion attributions predict externalizing behavior problems or that CU-traits and externalizing behavior are reciprocally and perhaps causally connected. Early antisocial behavior may predict increased callousness, manipulativeness, and grandiosity, implying a reciprocal or reverse impact from antisocial to externalizing behavior (Forsman et al., 2010). Sijtsema et al. (2019) explain the significant cross-lagged associations from externalizing behavior on CU-traits by the fact that the externalizing behaviors could emotionally desensitize youth. The non-significant effect of CU-traits on externalizing behavior problems in adolescents is also reported by Muratori et al. (2017). Emotional desensitization due to externalizing behavioral problems can also be applied to the present results. The desensitization of emotions speaks to both the effect of externalizing behavior problems on CU-traits and externalizing behavior problems on moral emotion attributions. A possible explanation for the discrepancies between theory and empiricism could be other uncontrolled variables like moral disengagement, which has previously been associated with CU-traits and externalizing behavior (Paciello et al., 2020). These results also imply, contrary to present theories of moral research, that morality does not have a predictive effect on behavior but, conversely, that behavior affects the development of moral constructs. A possible reason for these results could be that the adolescents who show externalizing behavior problems learned that they do not feel bad after rule violations, and thus they cannot transfer moral values into their identity. Given the body of research it could be shown, contrary to the present study results, that high moral identity is negatively related to the attribution of moral emotions and that these predict behavior. This is because the higher the moral identity, the worse adolescents feel about their selfish decisions (Schipper & Koglin, 2021b; Stets & Carter, 2011). The direction also contradicts the theoretical assumptions of Oser (2013) in which he states that moral action can be derived from moral emotions and moral identity. Analysis revealed high stability of externalizing behavior problems; therefore, it can be assumed that this formation can only occur when externalizing behavior problems are not yet manifest.

The additional analysis shows that all variables within each time were positively correlated with each other, excluding externalizing behavior problems_T2_ with moral identity_T1_, moral identity_T2_ and moral emotion attributions_T1_. The significant correlations found are consistent with the hypotheses that higher CU-traits for both test time points are associated with lower moral identity. Furthermore, CU-traits are found to be associated with better feelings after rule violations (higher moral emotion attributions) and higher externalizing behavior at both test time points. In addition, higher moral identity was expected to be associated with worse feelings after rule violations and fewer externalizing behavior problems at both test time points. The hypothesized correlations could be shown, with the exception of the correlations between moral identity at both test time points and externalizing behavior problems at both test time points. Correlative analysis for demographic variables only shows correlations with age and externalizing behavior problems for both time points. Externalizing behavior problems are also shown to increase with age. Gender is associated with moral identity for the first time point. Accordingly, girls are more often attributed with a high moral identity. The other variables in this study show no significant relationships with age and gender. When all variables in the ANOVA with repeated measurements are controlled for age and gender, no significant effects are found (Table 3). These findings are not consistent with the current body of research describing moral identity, CU-traits, and externalizing behavior problems to the detriment of boys (Bleyer et al., 2017; Fragkaki et al., 2016; Hardy, 2006; Hardy et al., 2014; Koglin & Petermann, 2012; Moylan et al., 2010).

### Limitations

In conclusion, the study contributes to the growing body of literature on CU-traits and morality with outcome behavior. Its limitations should be noted. This study was exploratory in nature, which is a primary limitation. In addition, there is a manageable sample size with low generalizability due to the ad-hoc sample. Future research is needed in order to replicate the findings in larger sample sizes. There is also a need for broader, transdisciplinary approaches that integrate different perspectives (e.g. biological) in conducting research for CU-traits (Forsman et al., 2010). Furthermore, it should be noted that the data was collected online through self-reports by adolescents. Despite these limitations, the study makes an important contribution to the literature of morality in dependence on personality factors which could be revealed by the method of stability and cross-lagged analysis.

### Implications

Furthermore, implications can be derived especially for future research. Due to the exploratory nature of the study, as well as the small number of studies considering the present constructs, focus should be on the replication of this study. Such a replication should consider a more comprehensive sample size as well as additional age groups. In particular, when considering younger age groups, it could be examined at what point the behavior itself begins to have an impact on moral emotion attribution and CU-traits. To pronounce implications for practice seems premature at this point. However, the inclusion of moral constructs seems promising when considering CU-traits and externalizing behavior problems in adolescents. The results show that adolescents’ behavior has an impact on moral emotion attributions. One possible reason for these results could be that the adolescents who exhibit externalizing behavior problems have learned that they do not feel bad after breaking rules and thus cannot transfer moral values into their identity, which in turn would make them feel bad. This may indicate that reinforcing moral values, emotions, and actions could be a possible module of future prevention and intervention.

## Summary

Even though the body of research has grown rapidly, very little is known about the interactions of adolescents callous-unemotional traits with moral constructs and the interplay of various outcomes. Moral identity is defined by the importance of moral qualities to the self-concept. Another part of the moral motivation for moral action are moral emotions attributions. CU-traits in childhood and adolescence are named as precursors of psychopathy in adulthood. They denote affective-social deficits such as low empathy, lack of remorse for wrongdoing, lack of emotional expression, and lack of concern about one’s own performance or the feelings of others. The present study builds on the lack of research and focuses on the longitudinal relationships between CU-traits, moral identity, moral emotion attribution and externalizing behavior problems in adolescence. The included variables were collected at test time points T1 and T2. Calculation of descriptive analysis, ANOVA with repeated measurement and MANOVA were conducted in SPSS Statistics version 27. To determine the predictive, and stability links among the variables, a cross-lagged model in SPSS AMOS 26 was conducted. Time stability path estimates were moderate to highly stable over time for all included variables. Significant cross lagged paths of moral identity_T1_ on moral emotion attribution_T2_, CU-traits_T1_ on moral identity_T2_, externalizing behavior problems_T1_ on moral emotion attributions_T2_ and externalizing behavior problems_T1_ on CU-traits_T2_, could be found.

In conclusion, the study contributes to the growing body of literature on CU-traits and morality with outcome behavior.

## Data Availability

The data that support the findings of this study are available from the corresponding author, upon reasonable request.
